# Characterization of Farmington virus, a novel virus from birds that is distantly related to members of the family *Rhabdoviridae*

**DOI:** 10.1186/1743-422X-10-219

**Published:** 2013-07-01

**Authors:** Gustavo Palacios, Naomi L Forrester, Nazir Savji, Amelia P A Travassos da Rosa, Hilda Guzman, Kelly DeToy, Vsevolod L Popov, Peter J Walker, W Ian Lipkin, Nikos Vasilakis, Robert B Tesh

**Affiliations:** 1 Center for Genomic Sciences, United States Army Medical Research Institute for Infectious Diseases, Frederick, MD 10032, USA; 2 Department of Pathology and Center for Biodefense and Emerging Infectious Diseases, University of Texas Medical Branch, Galveston, TX 77555-0609, USA; 3 Center for Tropical Diseases, University of Texas Medical Branch, Galveston, TX 77555-0609, USA; 4 Institute for Human Infection and Immunity, University of Texas Medical Branch, Galveston, TX 77555-0610, USA; 5 Center for Infection and Immunity, Mailman School of Public Health, Columbia University, New York, NY 10032, USA; 6 CSIRO Animal, Food and Health Sciences, Australian Animal Health Laboratory, Geelong, VIC 3220, Australia; 7 Current address: School of Medicine, New York University, New York, NY 10032, USA

**Keywords:** Farmington virus (FARV), Family *Rhabdoviridae*, Next generation sequencing, Phylogeny

## Abstract

**Background:**

Farmington virus (FARV) is a rhabdovirus that was isolated from a wild bird during an outbreak of epizootic eastern equine encephalitis on a pheasant farm in Connecticut, USA.

**Findings:**

Analysis of the nearly complete genome sequence of the prototype CT AN 114 strain indicates that it encodes the five canonical rhabdovirus structural proteins (N, P, M, G and L) with alternative ORFs (> 180 nt) in the N and G genes. Phenotypic and genetic characterization of FARV has confirmed that it is a novel rhabdovirus and probably represents a new species within the family *Rhabdoviridae.*

**Conclusions:**

In sum, our analysis indicates that FARV represents a new species within the family *Rhabdoviridae.*

## Background

The rhabdoviruses are a large and diverse group of single-stranded, negative sense RNA viruses that infect a wide range of vertebrates, invertebrates and plants [1]. The family *Rhabdoviridae* is currently divided into nine approved genera *(Vesiculovirus, Perhavirus, Ephemerovirus, Lyssavirus, Tibrovirus, Sigmavirus, Nucleorhabdovirus, Cytorhabdovirus* and *Novirhabdovirus)*; however, a large number of animal and plant viruses that appear to be members of the family have not yet been characterized or approved as species ([1]; http://www.ictvonline.org). Farmington virus (FARV) was isolated from an unidentified wild bird in 1969 during investigation of an epizootic of Eastern equine encephalitis on a pheasant farm in Farmington, Connecticut, USA [2]. The current report describes the phenotypic and genetic characterization of FARV, indicating that it is a novel rhabdovirus, which is very distantly related to other members of the family *Rhabdoviridae.*

## Results

### Growth characteristics

**Figure 1:**
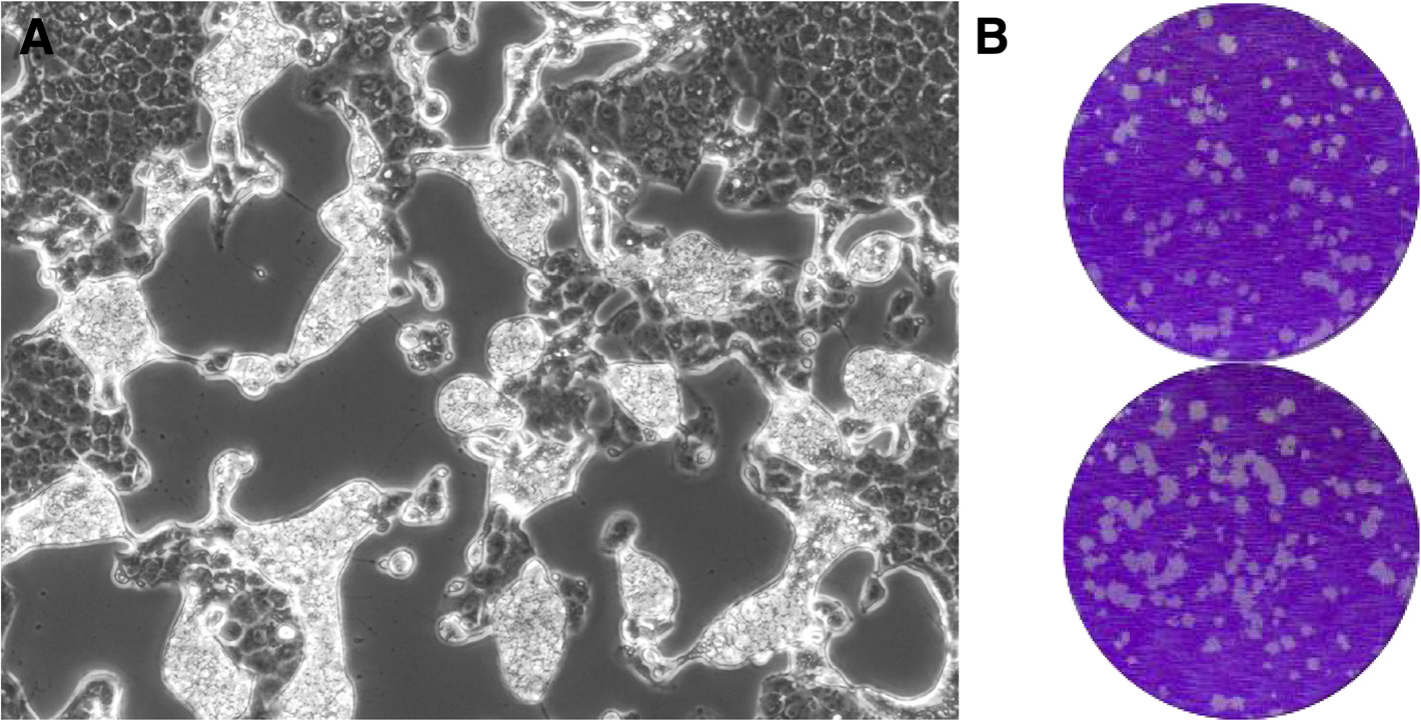
**Cytopathic effects of FARV virus infection. A**. Cytopathic effect produced by FARV in a Vero cell monolayer, 24 hrs after inoculation. Cells appear to detach from the surface of the culture flask and roll up in clumps. 100× magnification. **B**. Plaques (2–4 mm in diameter) produced by FARV in Vero cells under an agarose overlay, 3 days after inoculation.

Three litters of newborn (1–2 day old) ICR mice with average size of 10 pups were inoculated intracerebrally (ic) with 15–20 μl, intraperitoneally (ip) with 100 μl or subcutaneously (sc) with 100 μl of a stock of Vero-grown FARV (CT AN 114) virus containing approximately 10^7^ plaque forming units (PFU) per ml. The animals were observed daily for illness and were euthanized when they became severely ill. Within 2 days, all members of the ic-inoculated group were dead or dying. The groups inoculated ip or sc developed signs of illness (loss of balance, paralysis, lethargy) more slowly, but all were gravely ill by day 7 and were euthanized. In contrast, adult mice inoculated ip with a 10% suspension of infected newborn mouse brain did not become ill but subsequently developed high titers of complement fixing (CF) antibodies in their sera. Inoculation of CT 114 into Vero cells likewise produced massive cytopathic effect (CPE) within 24–48 h. Figure 1A and 1B show the CPE observed in Vero cells with fluid medium and the plaques produced under an agarose overlay, respectively. The growth of FARV in various vertebrate cell lines was described previously [3].

### Ultrastructure

**Figure 2:**
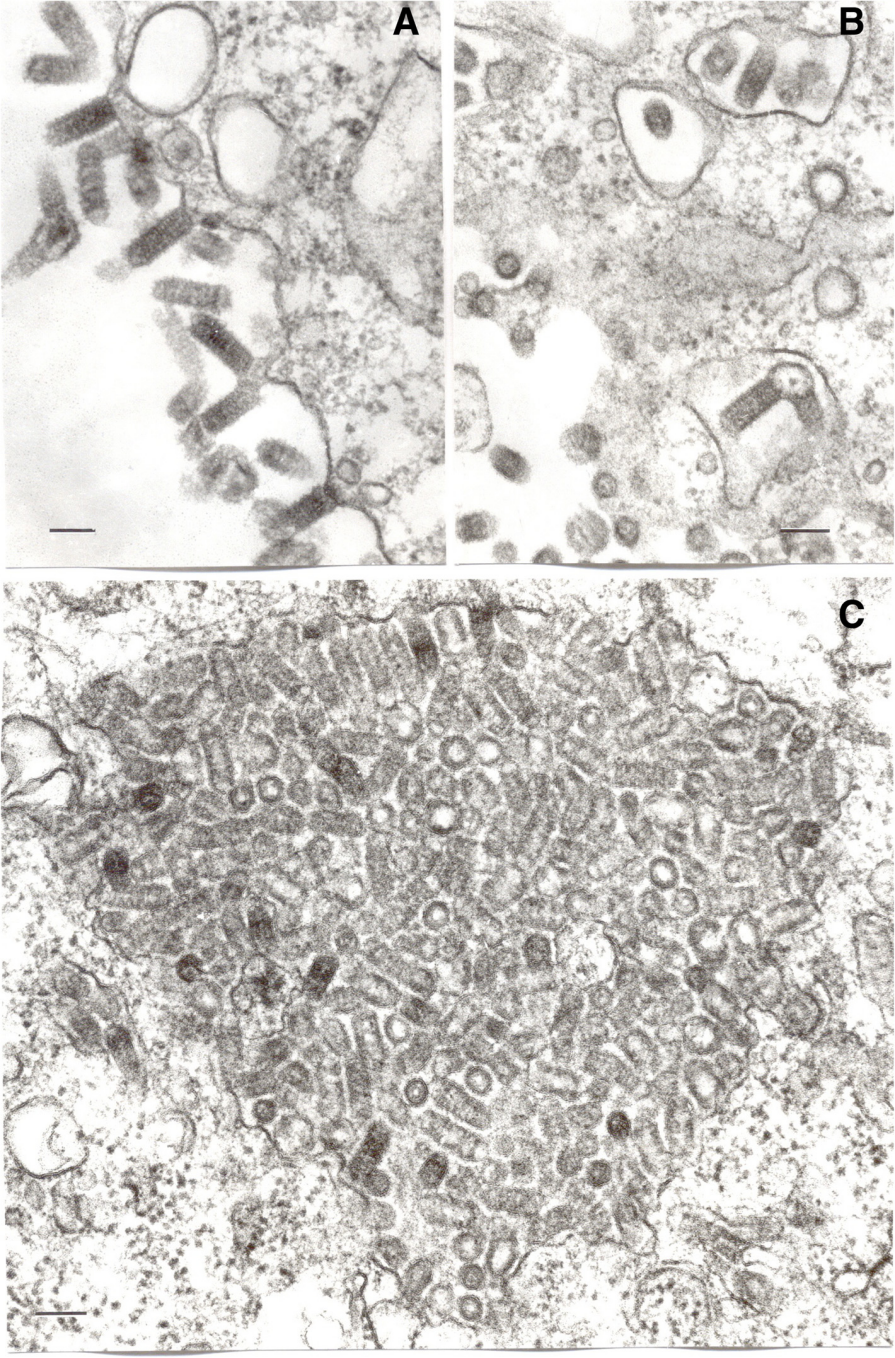
**Ultrastructure of FARV in Vero cells.** Bars = 100 nm. **A**- Virions forming at the cell surface. **B**- Budding of the virions into intracytosolic vacuoles. **C**- Large accumulation of virions in an intracytosolic vacuole.

In ultrathin sections of infected Vero cells, bullet-shaped virions 55–60 nm in diameter and 140–160 nm long were observed budding either from cell surface (Figure 2A) or into intracytosolic vacuoles (Figure 2B). Occasionally, these vacuoles contained large accumulations of virus particles (Figure 2C).

### Serology

**Figure 3:**
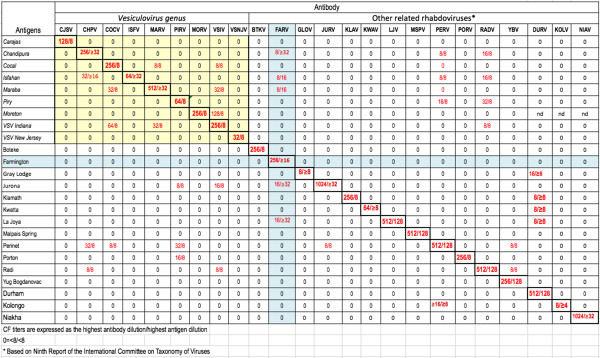
**Antigenic relationships of FARV and other selected rhabdoviruses, as determined by complement fixation tests.** Yellow shading highlights the antigenic relationships of members of the genus *Vesiculovirus,* whereas, light green shading highlights the antigenic relationships of FARV with other selected rhabdoviruses of this panel.

In cross-CF tests with other selected rhabdovirus antigens and antibodies, FARV mouse immune ascetic fluid antibody (MIAF) reacted with its homologous antigen to a dilution of 1:256; however, it also reacted at lower dilution (1:8 – 1:16) with chandipura virus (CHPV), Ishahan virus (ISFV), Maraba virus (MARV), Jurona virus (JURV) and LaJoya virus (LJV) antigens (Figure 3). CHPV, ISFV and MARV are currently designated as species in the genus *Vesiculovirus*, while JURV and LJV are listed as possible species in the same genus [1].

### Genome organization

**Figure 4:**
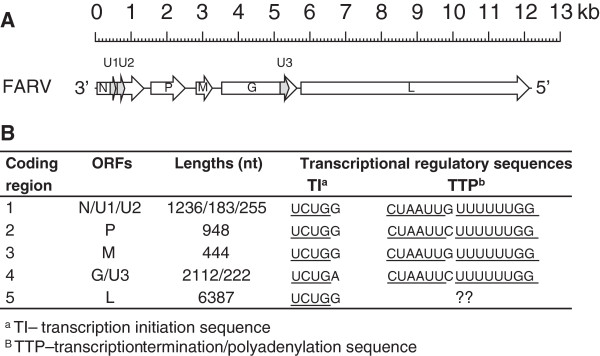
**Organization of FARV single-strand negative-sense RNA genome. (A)**. Schematic representation of the genome organization of FARV. **(B)**. FARV coding regions, designated ORFs and putative transcription regulatory sequences.

High throughput sequencing and assembly yielded a 12,146 nt contig that was identified as representing the FARV genome. The precise 3′- and 5′ -termini of the FARV genome were characterized by RACE yielding a complete genome sequence of 12,233 nt. Five coding regions were identified, each bounded by relatively conserved sequences that are likely to contain motifs that serve as transcription initiation (UCUG[G/A]) and transcription termination/polyadenylation (CUAA[A/U]U[G/C]UUUUUUGG) sequences. The five coding regions each contain a long ORF corresponding to the canonical rhabdovirus genomic organization 3′-N-P-M-G-L-5′ (Figure 4). Alternative ORFs encoding potential proteins of > 60 aa were also present in the putative N gene (2 consecutive ORFs, 183 nt and 255 nt) and the G gene (one ORF, 222 nt); however, only the second alternative ORF in the N gene featured an initiation codon in suitable context for translation [4]. Each of the five coding regions also a featured relatively long 3′-UTR (56–111 nt) and 5′-UTR (48–208 nt). There were no additional genes interposed between the major structural protein genes, as found in some genera of the *Rhabdoviridae*[5].

The 1236 nt FARV N ORF encodes an acidic (pKa = 5.2) 45.1 kDa protein that in pairwise alignments shares little sequence identity with the N proteins of other rhabdoviruses. BLASTp searches at NCBI and UniProt found no homology to any viral proteins. In addition, searches using the SMART software [6,7] revealed no conserved domains within the protein. However, Clustal X pair-wise sequence alignments indicated a high level of overall sequence similarity with vesiculovirus N proteins. For example, FARV N protein shares 41.5% and 39.3% similarity (identical or highly conservative amino acid substitutions) with the N proteins of Isfahan and Chandipura viruses, respectively (not shown).

The 951 nt FARV P ORF encodes a 35.8 kDa protein that shares no detectable homology with rhabdovirus P proteins, shares no significant identity with any characterized sequence when subjected to a BLASTp search, and no conserved domains were identified. However, like other rhabdovirus P proteins (which generally share quite low levels of sequence identity), the putative FARV P protein features a high proportion of charged residues (~32%), including various clusters in regions that are predominantly acidic or basic.

The 441 nt FARV M ORF encodes a mildly acidic (pKa = 9.23) 16.4 kDa protein that again shares no significant identity with any other known proteins. However, the N-terminal domain does contain the late budding domain motif (L-domain) PPxY that is present in many other rhabdovirus M proteins and is involved in the recruitment of cellular proteins involved in the budding process [8-14].

The 2115 nt FARV G ORF encodes a 704 aa protein for which a BLASTp search indicated no significant sequence homology to other proteins. However, pair-wise sequence alignments indicated that it shares some limited sequence similarity and common structural characteristics with other animal rhabdovirus G proteins. Like other G proteins, it is predicted to be class I transmembrane glycoprotein with an N-terminal signal peptide, heavily glycosylated ectodomain, a transmembrane domain and short C-terminal cytoplasmic domain. The ectodomain contains 12 potential N-glycosylation sites of which seven are predicted to be in a suitable context for glycosylation (http://www.cbs.dtu.dk/services/NetNGlyc/), indicating the mature G protein has a predicted size of ~102 kDa, which is somewhat larger that the G proteins of most other known rhabdoviruses. There are 16 cysteine residues in the ectodomain, many of which appear to align with 12 highly conserved cysteine residues (C_I_ – C_XII_) that occur commonly amongst animal rhabdovirus G proteins, forming 6 intramolecular disulphide bridges [11,14-17]. However, the FARV G protein does display some striking variations in the alignment of the conserved cysteines, particularly in the fusion domain (encompassing C_II_-C_IV_ and C_III_-C_V_ disulphide bridges and the C_VI_-C_VII_ loop), suggesting the folded structure may differ significantly from that of other G proteins (Additional file 1: Figure S1).

The 6390 nt FARV L ORF encodes a 234 kDa protein that has identifiable sequence homology to other rhabdovirus L proteins, containing all six conserved regions (CRs) and associated motifs associated with P protein binding (CRI), RNA template binding (CRII), RNA-dependent RNA polymerase (CRIII), polynucleotide transferase (CRV) and 2-O-methyl transferase activities [18,19]. In pairwise sequence alignments, the FARV L protein was found to be most closely related to viruses from the genus *Cytorhabdovirus*; FARV showed a maximum identity of 31% with the cytorhabdoviruses and a minimum identity of 26% with all other members of the *Rhabdoviridae*.

### Protein size as determined by immunoblotting

**Figure 5:**
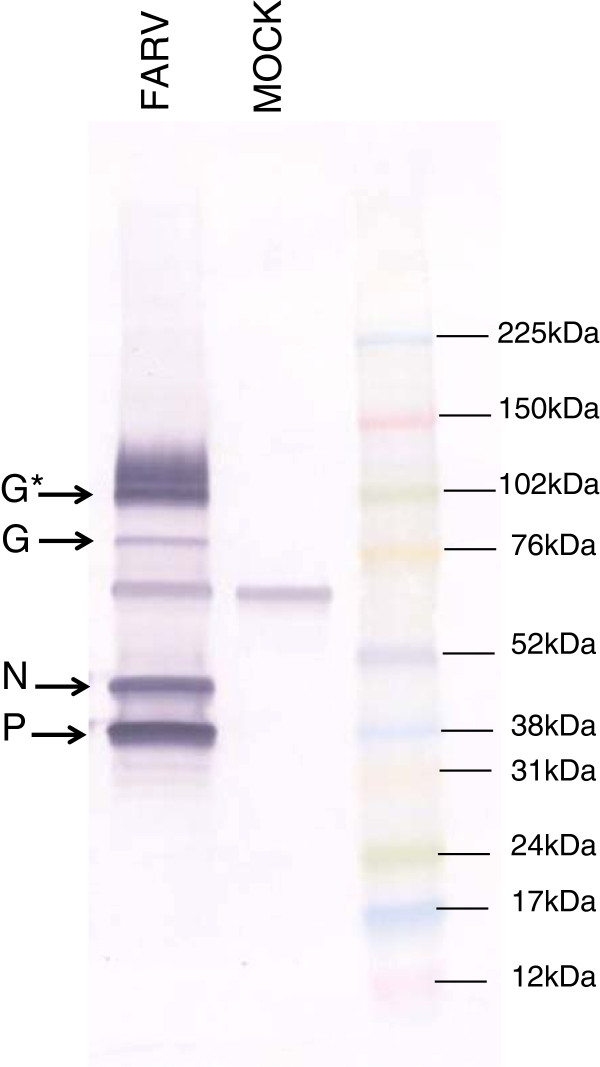
**Immunoblotting of FARV- and mock-infected Vero cells at four days post infection using mouse ascitic fluid made to FARV.** Proteins unique to FARV-infected cells are designated by protein abbreviations. A diffuse band (G*) corresponds to the predicted size for the mature G protein and a smaller sharper band (G) corresponds to the pre-processed, non-glycosylated form of the G protein. The molecular mass landmarks of the Rainbow Molecular Weight Marker® (GE Healthcare Life Sciences) are indicated on the right.

Viral proteins expressed in FARV-infected cells were analyzed by western immunoblotting. Extracts of FARV-infected and mock-infected Vero cells were separated by SDS-PAGE, transferred to a PVDF membrane and probed with the FARV mouse immune ascitic fluid (MIAF). At 2 days post-infection (dpi) four proteins were expressed differentially in infected cells (Figure 5). A diffuse band at ~102 kDa appeared to correspond in size to that predicted for the mature G protein and a smaller sharper band corresponded approximately in size (77.4 kDa) to the pre-processed, non-glycosylated form of the G protein. Bands at ~45 kDa and ~38 kDa corresponded in size to those predicted for the FARV N and P proteins, respectively. The ~38 kDa band appeared to migrate as a doublet with a second slightly smaller band which may be alternatively phosphorylated as has been reported for the rabies virus P protein [20]. There was no evidence of detection with this MIAF of the L or M proteins or of lower molecular weight proteins predicted to be encoded in the alternative ORFs.

### Phylogeny

**Figure 6:**
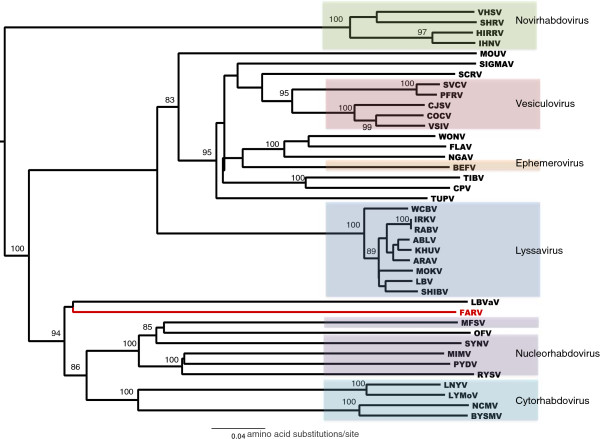
Neighbor-joining phylogenetic tree produced using a concatenated alignment of G-Blocks.

Due to the lack of extensive amino acid sequence identity between FARV and the other rhabdoviruses, a concatenated section of the conserved domains of the L protein was utilized in which all ambiguously aligned regions were removed using the G-blocks program (Figure 6) [21]. The placement of FARV was problematic as it grouped with high bootstrap support with the genera *Nucleorhabdovirus* and *Cytorhabdovirus*; however, there was no significant support for the placement of FARV within these genera of plant rhabdoviruses. FARV also grouped closely with lettuce big-vein associated virus (LBVaV) but this had no significant bootstrap support. LBVaV is currently classified as a member of the unassigned genus *Varicosavirus* and is distantly related to the *Rhabdoviridae*[22]. Due to the low level of sequence identity in the L proteins, the deep evolutionary roots of these viruses remain obscure. Moreover, the L proteins are conserved across many negative stranded RNA viruses, including members of the order *Mononegavirales*, and of the families *Bunyaviridae* and *Arenaviridae*, suggesting that they may share a common ancestor. While the gene organization clearly indicates that FARV is a rhabdovirus, the genetic distance and lack of identifiable sequence homology between FARV and the rest of the well-characterized *Rhabdoviridae* suggests that it probably represents the foundation member of a new genus. The most striking observation of this analysis is that FARV showed high bootstrap support for placement in a clade that only includes non-mammalian *Rhabdoviridae.*

## Discussion and conclusions

The family *Rhabdoviridae* is a very diverse taxon, which includes viruses that infect a wide range of hosts. Due to the genetic diversity within the *Rhabdoviridae*, it has been difficult to identify evolutionary relationships between and within the most diverse groups. In addition, this has been complicated by viruses such as LBVaV which, although differing in morphology and genome organization, has similarities in their in the amino acid sequences of both the nucleoprotein (N protein) coding regions and RNA-dependent RNA polymerase (L protein) with those of rhabdoviruses [22].

When FARV was first isolated in 1969, it could not be identified and was deposited in the World Reference Center for Emerging Viruses and Arboviruses (WRCEVA) collection as an unknown virus. Later ultrastructural and serologic studies [2] indicated that it was a rhabdovirus, possibly a vesiculovirus. Based on the serologic results shown in Figure 3, FARV appears to have a distant relationship to several viruses in the genus *Vesiculovirus* as reported previously [2]. The re-examination of FARV by deep sequencing presented here indicates that the genome organization and ultrastructure are consistent with its classification as a rhabdovirus (Figures 2 and 4), although the relationship to the members of the genus *vesiculovirus* cannot be correlated with any sequence similarity. Our sequence analysis indicates little overall identity of FARV to the other known rhabdoviruses in most proteins, except for the polymerase. Immunoblot analysis employing the FARV MIAF that was used in CF tests indicated the major homologous reactions were with the G, N and P proteins. It is likely, therefore, that antigenic cross-reactions detected against vesiculoviruses in CF tests are due to common epitopes in one or other of these proteins. Indeed, pair-wise sequence alignments of the FARV N and P proteins with the corresponding vesiculovirus proteins did indicate several short stretches of amino acid sequence (8–9 amino acids) in with the sequence identity is high (not shown). Similar limited regions of high identity in the N protein have been reported previously to account for distant antigenic cross-reactions between the ephemeroviruses and lyssaviruses [23].

One of the major issues with using sequences as a means to determine relationships within the *Rhabdoviridae* is that the distance between the various groups is so large that any nucleotide alignment would not be any different from random, thus nucleotide alignments do not give a robust construction of the relationships between the different genera. Because of this, the global relationships across the *Rhabdoviridae* have previously been determined by using the RNA-dependent RNA polymerase domain of L gene protein sequence [24]. Even so, only a few regions exhibit a sufficiently robust alignment to allow a reconstruction of the relationships. While we have utilized this approach to infer the relationship between FARV and other members of the family *Rhabdoviridae*, there are insufficient sequences available in the public domain (e.g., GenBank) to determine their deep evolutionary relationships.

Genetically, FARV is also similar to the varicosavirus LBVaV. Varicosaviruses are negative-sense ssRNA viruses that have two linear RNA segments and infect plants (lettuce and tobacco) [25]. As noted above, the varicosaviruses have some similarities in genome structure and probable transcription mechanism with the rhabdoviruses but appear to lack a G gene homologue and have filamentous particles that resemble rhabdovirus nucleocapsids. For this reason, LBVaV was included in our phylogenetic analysis (Figure 6). Structurally and phylogenetically, varicosaviruses are distantly related to *Rhabdoviridae* but the evolutionary history and host adaptation processes that have led to genome segmentation and gene loss is less clear. An alternative tree in which the LBVaV was excluded, under the assumption that varicosaviruses may not be truly related to *Rhabdoviridae,* did not result in any different placement of FARV, nor to its bootstrap support.

The phylogenetic position of FARV is intriguing. It sits basal to the two plant rhabdovirus genera, *Nucleorhabdovirus* and *Cytorhabdovirus* along with LBVaV, unassigned genus *Varicosavirus* (Figure 6). Yet, it was originally isolated from a bird and clearly infects mammals and mammalian cells. Given these characteristics, could FARV be a derivative of an ancient plant rhabdovirus that has adapted to its niche through genome segmentation and loss of genes that it no longer required for replication and transmission? Unfortunately, because of the lack of alignment confidence and the genetic distance between the various genera, it is not possible to resolve the evolutionary history of the rhabdoviruses at this time.

## Methods

### Virus

The prototype strain of FARV (CT AN 114) was obtained from the World Reference Center for Emerging Viruses and Arboviruses (WRCEVA) at the University of Texas Medical Branch. As noted previously, CT AN 114 was isolated from an unidentified bird collected during an epidemiologic investigation of an outbreak of eastern equine encephalitis on a commercial pheasant farm in Farmington, CT in 1969. At the time of isolation, it could not be identified and was deposited in WRCEVA virus collection as an unknown. The virus strain used in our studies had been passaged 11 times by intracerebral inoculation of newborn mice and twice in Vero cells. The virus used for sequencing was prepared from a single plaque picked from a monolayer culture of Vero cells.

### Cell culture

Vero E6 cells (African green monkey kidney) were used for propagating the virus. The cells were originally obtained from the American Type Culture Collection (Manassas, VA) and were grown at 37°C in minimal essential medium (MEM) with Earle’s salts (Gibco/Invitrogen, Carlsbad, CA) supplemented with 5% heat-inactivated (56°C for 30 min) fetal bovine serum (FBS) and 1% penicillin-streptomycin stock (Sigma, St. Louis, MO).

### Animals

Outbred ICR mice (Harlan Sprague–Dawley, Indianapolis, IN) were used to prepare antigens and antibodies for FARV and the other rhabdoviruses shown in Figure 3. All animal work was carried out under a protocol approved by the University of Texas Medical Branch Institutional Animal Care and Use Committee.

### Antigens and immune reagents

Antigens used in CF tests were prepared from infected newborn mouse brains extracted by the sucrose/acetone method [26]. Specific mouse immune ascitic fluids (MIAF) were also prepared against each of the 24 rhabdoviruses listed in Figure 3. Immunogens were 10% suspensions of homogenized infected mouse brain in phosphate-buffered saline mixed with complete Freund’s adjuvant. The immunization schedule consisted of four intraperitoneal injections given at weekly intervals. Sarcoma 180 cells were given intraperitoneally with the final immunization in order to induce ascites formation.

### Serologic tests

CF tests were done according to a microtechnique described previously [27,28], using 2 full units of guinea pig complement. Titers were recorded as the highest dilutions giving 3+ or 4+ fixation of complement on a scale of 0 to 4 + .

### Transmission electron microscopy

For ultrastructural analysis, infected Vero cells were fixed for at least 1 h in a mixture of 2.5% formaldehyde prepared from paraformaldehyde powder, and 0.1% glutaraldehyde in 0.05 M cacodylate buffer, pH 7.3, to which 0.03% picric acid and 0.03% CaCl_2_ were added. The infected cell monolayers were washed in 0.1 M cacodylate buffer, scraped off, and processed further as a pellet. The pellets were post-fixed in 1% OsO_4_ in 0.1 M cacodylate buffer, pH 7.3, for 1 h, washed with distilled water, and stained *en bloc* with 2% aqueous uranyl acetate for 20 min at 60°C. The pellets were dehydrated in ethanol, processed through propylene oxide and embedded in Poly/Bed 812 (Polysciences, Warrington, PA). Ultrathin sections were cut on a Leica EM UC7 ultramicrotome (Leica Microsystems, Buffalo Grove, IL), stained with lead citrate and examined in a Phillips 201 transmission electron microscope at 60 kV.

### Immunoblotting

Vero monolayers were grown to 90% confluency in 25 cm^2^ plastic tissue culture flasks and were infected with FARV or were mock infected. Virus inocula were aspirated following 1 h of absorption at 37°C, washed thrice to remove any unabsorbed virus, replaced with fresh medium (MEM) supplemented with 5% FBS and 1% penicillin/streptomycin, and incubated at 37°C. Four days post infection (p.i.), the cells were harvested into cell lysis buffer (50 mM Tris–HCl, pH 8.0, 150 mM NaCl, 1% Triton X-100). The cell preparations were then treated according to the Biorad instructions for MiniPROTEAN TGX gel analysis of proteins. Briefly, 20 μl samples of loading buffer containing β-mercaptoethanol were added to 20 μl aliquots of the cell lysates. Samples were heated at 95°C for 10 min, and centrifuged for 1 min at 500 × *g* at 4°C. Proteins were separated under reducing conditions on MiniPROTEAN TGX 4-20% Tris-Glycine SDS-PAGE gels, and were transferred onto Hybond-P PVDF membrane (Amersham) with Transfer Buffer (Biorad, Hercules, CA) according to the manufacturer’s western blotting transfer protocol. Membranes were processed by the WesternBreeze© Chromogenic Immunodetection system (Invitrogen), following the manufacturer’s protocol. The polyclonal MIAF raised to FARV was used at 1:5000 dilution.

### Nucleotide sequence accession numbers

The genome sequence of FARV was determined in this study and assigned GenBank accession number KC602379. The GenBank accession numbers for the genome sequences of select rhabdoviruses used in the phylogenetic analyses are listed as follows: ARV, Ade laide River virus (AFR23540); ABLV, Australian bat lyssavirus (NP478343); ARAV, Aravan virus (ABV03822); BEFV, bovine ephemeral fever virus (NP065409); CHPV, Chandipura virus (AED98393); COCV, Cocal virus (ACB47438); CPV, Coastal Plains virus (ADG86364); DMelSV, *Drosophila megalomaster* sigmavirus (YP003126913); DObSV, *Drosophila obscura* sigmavirus (ACU65444); DURV, Durham virus (ADB88761); DUVV, Duvenhage virus (AFK93192); EVEX, eel virus European X (AFX58972); EBLV1, European bat lyssavirus 1 (YP001285392); FLAV, Flanders virus (AAN73288); HIRV, Hirame rhabdovirus (NP919035); IKOV, Ikoma lyssavirus (AFQ26098); IHNV, infectious hematopoietic necrosis virus (NP042681); IRKV, Irkut virus (ABV03823); ISFV, Isfahan virus (CAH17548); JURV, Jurona virus (AEG25349); KHUV, Khujand virus (ABV03824); KIMV, Kimberley virus (AFR67096); KOTV, kotonkan virus (YP006202628); LBV, Lagos bat virus (AFW16650); MARV, Maraba virus (AEI52253); MOKV, Mokola virus (YP142354); MOUV, Moussa virus (ACZ81407); NGAV, Ngaingan virus (YP003518294); OVRV, Oak Vale rhabdovirus (AEJ07650); OBOV, Obodhiang virus (YP0062000965); OZEV, Ozernoe virus (ACS70797); PRV, Perinet virus (AEG25355); PFRV, pike fry rhabdovirus (ACP28002); RABV, rabies virus (ABN11300); SHIBV, Shimoni bat virus (ADD84511); SCRV, *Siniperca chuatsi* rhabdovirus (YP802942); SHRV, snakehead virus (NP050585); SVCV, spring viraemia of carp virus (NP116748); TIBV, Tibrogargan virus (ADG86355); TUPV, tupaia virus (YP238534); VSAV, vesicular stomatitis Alagoas virus (ACB47443); VHSV, viral hemorrhagic septicemia virus (BAM29126); VSIV, vesicular stomatitis Indiana virus (NP041716); VSNJV, vesicular stomatitis New Jersey virus (AAA48442); WCBV, West Caucasian bat virus (ABV03821); and WONV, Wongabel virus (YP002333280). The ICTV has not yet recognized FARV as a member species in the family *Rhabdoviridae*. As a consequence, its abbreviation ought to be considered tentative and subject to approval.

### Genome sequencing

RNA was extracted from virus stocks (infected Vero cells) using TRIzol LS (Invitrogen) and treated with DNase I (DNA-Free, Ambion, Austin, TX). cDNA was generated using the Superscript II system (Invitrogen) employing random hexamers linked to an arbitrary 17-mer primer sequence [29], treated with RNase H and then randomly amplified by PCR with a 9:1 mixture of primer corresponding to the 17-mer sequence and the random hexamer linked 17-mer primer. Products greater than 70 base pairs (bp) were selected by column chromatography (MinElute, Qiagen, Hilden, Germany) and ligated to specific adapters for sequencing on the 454 Genome Sequencer FLX (454 Life Sciences, Branford, CT) without fragmentation [30,31]. After primer removal, redundancy filtering, and sequence assembly, sequence gaps were completed by RT-PCR amplification, using primers based on pyrosequencing data. Amplification products were size-fractioned on 1% agarose gels, prufied (MiniElute, Qiagen) and directly sequenced in both directions with ABI PRISM Big Dye Terminator 1.1 Cycle Sequencing kits on ABI PRISM 3700 DNA Analyzers (Perkin-Elmer Applied Biosystems, Foster City, CA). The terminal sequences for each genome were amplified using the Clontech SMARTer RACE kit (Clontech, Mountain View, CA). Genome sequences were verified by Sanger dideoxy sequencing using primers designed from the draft sequence to create products of 1,000 bp with 500 bp overlaps.

### Rapid amplification of cDNA ends (RACE)

Genomic termini were characterized with 5_- and 3_-RACE kits (Invitrogen). Virus-specific primers for FARV were: 5′ – GTC TTG AAG TCG TTT CCC AG, located 934 nt from the 3′ -genomic terminus for reverse transcription, 5′ – TCA GGT TCA TCA GCC ATT TC, located 618 nt from the 3′ – genomic terminus for first PCR with primer UAP (Invitrogen), and 5′ – ACC AGC CGA TGA TGT AAG C, located 556 nt from the 3′ – genomic terminus for second PCR with primer AUAP (Invitrogen). RNA for the second RACE was tailed with poly(A) polymerase (Ambion) and purified using RNeasy® Mini kit (Qiagen). cDNA synthesis was primed with oligo d(T) – adapter primer AP (Invitrogen), and first PCR used primer 5′ – AAC CGT TCC TTC ACT ACA TC, located 1,004 nt from the 3′– antigenomic terminus and primer UAP (Invitrogen), the second PCR used primer 5′ – TCG CTT ACC AGC ATT TTG AG, located 746 nt from the 3′ – antigenomic terminus and primer AUAP (Invitrogen). All primers were at 0.2 M final concentration. PCR products were purified with QIAquick PCR purification kits (Qiagen) and directly dideoxy-sequenced in both directions.

### Phylogenetic analysis

The L protein sequence of FARV was compared with those of 38 other rhabdoviruses downloaded from GenBank. All protein sequences were aligned using MUSCLE [32] under default settings. Because these sequences were highly divergent, which could negatively impact phylogenetic analysis, all ambiguously aligned regions were removed using the G-blocks program [21]. This resulted in a final sequence alignment of 433 amino acid residues. The phylogenetic relationships among these sequences were determined using the maximum likelihood (ML) method available in PhyML 3.0 [33] employing the WAG-G model of amino acid substitution and subtree pruning and regrafting (SPR) branch-swapping. The robustness of each node was evaluated using the SH and ChiSq minimum statistic. In addition, trees were determined using the neighbor-joining (NJ) and maximum parsimony (MP) methods using the default settings available in PAUP* v. b10, bootstraps were run for both methods.

## Abbreviations

FARV: Farmington virus; CF: Complement fixation; CPE: Cytopathic effect; MIAF: Mouse immune ascitic fluid; CHPV: Chandipura virus; ISFV: Isfahan virus; MARV: Maraba virus; JURV: Jurona virus; LJV: LaJoya virus; ORF: Open reading frame; dpi: Days post-infection; LBVaV: Lettuce big-vein associated virus; WRCEVA: World reference center for emerging viruses and arboviruses; MEM: Minimal essential medium; FBS: Fetal bovine serum.

## Competing interests

The authors do hereby declare that they have no competing interests in this scientific work.

## Authors’ contributions

GP, NS, NV, KdT, HG, RBT performed the laboratory experiments. APATdR performed the serologic assays. NFL performed the phylogenetic analyses; VP performed electron microscopy; PJW performed the genomic analyses. NV, PJW, NFL, WIL, CP, RBT contributed to final the manuscript preparation. All authors have read and approved the final manuscript.

## Supplementary Material

Additional file 1: Figure S1 Amino acid sequence alignment of FARV and 13 other rhabdovirus G proteins representing approved genera *Lyssavirus* (RABV- *Rabies virus*), Vesiculovirus (*Vesicular stomatitis Indiana virus* – VSIV), *Ephemerovirus* (*Bovine ephemeral fever virus* – BEFV), *Novirhabdovirus* (*Infectious hematopoietic necrosis virus* – IHNV), Tibrovirus (*Tibrogarban virus* – *TIBV*), *Sigmavirus* (*Drosophila melanogaster sigmavirus* – DmSv), unassigned species *Wongabel virus* (WONV), *Ngaingan virus* (NGAV), *Flanders virus* (FLAV), *Tupaia virus* (TUPV) and *Moussa virus* (MOUV) and presently unclassified viruses Durham virus (DURV) and Niakha virus (NIAV). The predicted N-terminal signal peptides and C-terminal transmembrane domains are shaded in aqua; conserved cysteine residues are shaded in grey; and other relatively conserved residues are shaded in black. Cysteine residues (dark shading) are numbered according to the conserved patterns observed in all animal rhabdovirus G proteins. Click here for file
