# Supplementary material for: Characterization of Farmington virus, a novel virus from birds that is distantly related to members of the family Rhabdoviridae
Source: Virol J. 2013 Jul 1;10:219. doi: 10.1186/1743-422X-10-219 (PMC3722107; doi:10.1186/1743-422X-10-219)
Supplement: Additional file 1: Figure S1 — Amino acid sequence alignment of FARV and 13 other rhabdovirus G proteins representing approved genera Lyssavirus (RABV- Rabies virus), Vesiculovirus (Vesicular stomatitis Indiana virus – VSIV), Ephemerovirus (Bovine ephemeral fever virus – BEFV), Novirhabdovirus (Infectious hematopoietic necrosis virus – IHNV), Tibrovirus (Tibrogarban virus – TIBV), Sigmavirus (Drosophila melanogaster sigmavirus – DmSv), unassigned species Wongabel virus (WONV), Ngaingan virus (NGAV), Flanders virus (FLAV), Tupaia virus (TUPV) and Moussa virus (MOUV) and presently unclassified viruses Durham virus (DURV) and Niakha virus (NIAV). The predicted N-terminal signal peptides and C-terminal transmembrane domains are shaded in aqua; conserved cysteine residues are shaded in grey; and other relatively conserved residues are shaded in black. Cysteine residues (dark shading) are numbered according to the conserved patterns observed in all animal rhabdovirus G proteins. [file 1743-422X-10-219-S1.docx]

**Supplementary Figure S1.** Amino acid sequence alignment of FARV and 13 other rhabdovirus G proteins representing approved genera *Lyssavirus* (RABV- *Rabies virus*), Vesiculovirus (*Vesicular stomatitis Indiana virus* – VSIV), *Ephemerovirus* (*Bovine ephemeral fever virus* – BEFV), *Novirhabdovirus* (*Infectious hematopoietic necrosis virus* – IHNV), Tibrovirus (*Tibrogarban virus* – *TIBV*), *Sigmavirus* (*Drosophila melanogaster sigmavirus* – DmSv), unassigned species *Wongabel virus* (WONV), *Ngaingan virus* (NGAV), *Flanders virus* (FLAV), *Tupaia virus* (TUPV) and *Moussa virus* (MOUV) and presently unclassified viruses Durham virus (DURV) and Niakha virus (NIAV). The predicted N-terminal signal peptides and C-terminal transmembrane domains are shaded in aqua; conserved cysteine residues are shaded in grey; and other relatively conserved residues are shaded in black. Cysteine residues (dark shading) are numbered according to the conserved patterns observed in all animal rhabdovirus G proteins [[1](#_ENREF_1)].

BEFV_G MFKVLIITLLVNKIHL--------------EKIY------------------------------------------------------------------

DURV_G MWIILLHVSFVAS-----------------QVI-------------------------------------------------------------------

FLAV_G MSYLIVKVVILFLVGIDKQVLS--------WTHDSGRSFVRQYHDPNWFDQTM-----------------------------------------------

IHNV_G MDTTITTPLILILITCGANS----------QTVKPDTASESDQPTWSNPLF-------------------------------------------------

NGAV_G MKSTILMTCLLLLHCTIHG-----------DIIPNFYNSYKIGQKQRRKLIYSKKEISSPPPPTTPPSNFILDDSEERKDGGMFTTDSVIPVGPVGGVSF

RABV_G MVPQALLFVPLLVFPLCFG-----------KFPI------------------------------------------------------------------

TUPV_G MAPQTISLLWAMVCVSVYTRA---------NRV-------------------------------------------------------------------

VSINV_G MKCLLYLAFLFIGVNC--------------KFTI------------------------------------------------------------------

WONV_G MSFLLAIIIFFFRTQDSNG-----------YVVQSGIGGIRESKVNILGQVVPYSTKFKHSVLKSQWDYRHDKGHRT-----------------------

TIBV_G MEAITIEIIIIILTISYPILVAP-------QLLY------------------------------------------------------------------

MOUV_G MRTLVIWVLINATMAFA-------------KPPGSASLSLGLY---------------------------------------------------------

DMeSV_G MVHYETHILFIHLWMLALIFITTSVWLAASQKTFTPDL--------------------------------------------------------------

NIAV_G MSQGYGLLFLVSGVLSLTAG----------YVF-------------------------------------------------------------------

FARV_G MLRIQIPPIAIILVSLLTLDLSGA------RRTTTQRIPLLNDSWDLFSS--------------------------------------------------

BEFV_G -----------------------------NVP-VNCG--------------------ELH---PVKAHEIKC--PQRLNELSLQAHHNLAKDEHYN----

DURV_G -----------------------------IAP-LREPQ-------------------EWR---VATKSDFKC-TP-GMLDDIEGPIDFW-----------

FLAV_G -----------------------------VYP-IECNS-------------------TWQ---EVNTLNLRC--PKSLKIDPKNKLNFDLG---------

IHNV_G -----------------------------TYP-EGCTL------------------DKLS---KVNASQLRC--PRIFDDENRGLIAYPTSIR-------

NGAV_G HNTKSQDTKVALVTAGTYNISDFNSHHLMNFP-VKCVG-------------------DWK---EVDLSSIRC--PRFSNLDMMNEGRREIG---------

RABV_G -----------------------------YTI-PDKLG-------------------PWS---PIDIHHLSC--PNNLVVEDEGCTNLSGFSY-------

TUPV_G -----------------------------VAP-IHEPQ-------------------NWK---PATVDDFTCRTG-FNLDFDSKFIKT------------

VSINV_G -----------------------------VFP-HNQKG-------------------NWK---NVPSNYHYC--PSSSDLNWHNDLIGTAL---------

WONV_G -----------------------------VLP-TNCHA-------------------TWN---DITPSNMRC--PRRKIIGKDGLYNTYIG---------

TIBV_G -----------------------------NYP-FNCK--------------------KGP---KMTLDGLTC--PLDFNTFNLDSKDNMEAG--------

MOUV_G -----------------------------WVPRIDNN--------------------TWK---SVHTTNLVC--PSFVGSVLPEMEESFEV---------

DMeSV_G -----------------------------VFPEMNRNS-------------------SWS---VANYGEILC--PTSFQSYDPKKHQILTRV--------

NIAV_G -----------------------------HYP-IEKDI-------------------HWY---PANHSSLRC--PIRSASITDTPTGGV-----------

FARV_G ------------------------------YGDIPEELVVYQNYSHNSSELPPPGFERWYINRRVADTSIPCRGPCLVPYILHGLNDTTVSRRGGGWRRS

**0 I a**

BEFV_G -------KICRPQLKDD---AHLEGFICRKQRWITKCSETWYFSTSIEYQILEVIPEYSGCTDAVKKLDQGA---LI-----PPYYPP------------

DURV_G -------RVSTIKADAN---LHIEGYACQAEIWISKCEETWYFSKTITHSVQFPEGTQHSCQDELRKQKAEG---AP-----VFRFPD------------

FLAV_G -------TVYHPLPSSR---YVVNGYICHKQTWISKCEETWYFSTTETNKIENVPITPEDCREAVTIYEMGE---YV-----NPFFPP------------

IHNV_G -------SLSVGNDLGE---IHTQGNYIHKVLYRTICSTGFFGGQTIEKALVEMKLSTKEAGA----YDTTT---AA-----ALYFPA------------

NGAV_G -------LAIHPVVSDG---VIAQGLLCQKQKWISECSETWYWTTEETSYVENEPVSGDECLLAYSQFKVGK---HI-----EPAFPP------------

RABV_G -------MELKVGYILA---IKVNGFTCTGVVTEAETYTNFVGYVTTTFKRKHFRPTPDACRAAYNWKMAGDPRYEE-----SLHNPY------------

TUPV_G -------KALVLKRVGQ---AKVKGYLCMKNRWTTTCETNWLYSKSVSHHITHVAVSAEECYNKIRDDASGN---LK-----IESYPN------------

VSINV_G -------QVKMPKSHKA---IQADGWMCHASKWVTTCDFRWYGPKYITHSIRSFTPSVEQCKESIEQTKQGT---WL-----NPGFPP------------

WONV_G -------DFWHPHTDKG---SEVKGFICQKTKWVSTCIETWYFSTTKETQIDEVPITKEDCLAAITLVDSGE---YI-----EPFFPP------------

TIBV_G -------TMCRPNPLSK---DIEDGFLCYKDTWVTTCEETWYFSKTVKNHIIHEHITKDECFEALATYKLGK---HV-----EPFFPA------------

MOUV_G -------DIQVPKHSQTT--SHQGGYLCYGFSFSVVCEEGFWGGQKVTEHTFTHLVSSEECLKAIEDKKSGE---YR-----PPHTPV------------

DMeSV_G -------LVERPSLNTD---TKVEGYTCHKVKYETICDMPWYFSPTISHSISPLRVKESECKDAIAEHQLGT---HV-----SLSFPP------------

NIAV_G -------TISIPSNPSN---NDLPGFSCHKTEWISECTETWYWSTDVKQYIRPVSVTADECKKAQRDKEVGT---EI-----TPFFTA------------

FARV_G GMKYPTHAVRLGPSTDDERVEEDIGYV---NVSALSCTGS---------PVEMAIPTIPDCTSAIHPRSEVTVPVKLDVMRRNPNYPPIRAWSCIGQKIT

**Ib** * **II III IV**

BEFV_G AGCFW---------NTEMNQEIEFYVLIQ----HKPFLN------PYDNLIYDS----RFLT-PCTINDSKTKGCPLKDITGTWIPDVRVEEISEHCNNK

DURV_G VECAY---------ASTTTATSYFLRLTP----MNVELN------PYASTLLHP----SFRDGKCTDLEV----CPMLHHHGLWIPKEPIAKTEEQAVFE

FLAV_G FYCSW---------SSTQIDKKTFVIVEP----HIVKED------IYNKTFIDP----FFLNGYCDQLP-----CKTIHPDVLWVPQELQKR-KDLCNKG

IHNV_G PRCQWY--------TDNVQNDLIFYYTTQ----KSVLRD------PYTRDFLDS----DFIGGKCTKSP-----CQTHWSNVVWMGDAG----IPACDSS

NGAV_G FACYW---------NSVNKVHQSYITLHD----HDVKLD------PYTDKFIDP----ILYGGQCEGSL-----CPTIHDNVYWIEKDDGED-LTICNLH

RABV_G PDYRWL--------RTVKTTKESLVIISP----SVADLD------PYDRSLHSR----VFPSGKCSGVAVSSTYCSTNHDYTIWMPENPRL--GMSCDIF

TUPV_G PQCAW---------SSTVSREEDFIHIST----SDVGYD------MYTDTVLSP----SFPGGTCKLKTC----CKTIYPNIVWVPETPAQ--TQVRDAL

VSINV_G QSCGY---------ATVTDAEAVIVQVTP----HHVLVD------EYTGEWVDS----QFIDGKCSNDI-----CPTVHNSTTWHSDYKV---KGLCDSN

WONV_G HVCSW---------ASTNKNSKEFVTVHE----HSVVLD------IYENKLMDP----IFLAGKCFDKV-----CKTIHRNVLWVEANDNER-DDFCVAT

TIBV_G PSCYW---------SATNEERATFVNIQP----HGVLLD------PYSGKIKDP----LIDSDNCDNDF-----CVTRSHQTHWLRNRKPDI-MERCNNE

MOUV_G SECGW---------MQTNTKTLRFVALEE----HPVLFD------PYTVNFVDG----LFEKTLCNQRI-----CPTVHANTIWIGDNEP---KKDCPST

DMeSV_G EDCSW---------NSVNTKAYEDIIVKD----HPVMLD------PYTNNYVDA----IFPGGISSP-----GMGGTIHDDMMWVSKDLAV--SPECSGW

NIAV_G PVCQW---------SNTVRKVNSFVITNK----KNVKFD------PYNLDFIDP----ILVGGRCKGNQES---CPTIQAGVIWLPR------LQPTKAT

FARV_G NRCDWALFGENLIYTQVEASSLAFKHTRASLLNESNGIDAEGRAVPYILGDIEP------------------GYCRTLFN--TWVSSEIVS-----CTPI

**V** *  **VI VII * VIII**

BEFV_G HWECITVKS--FRSELNDKERL--------------WEAPDIGLVHV--NKGCLS-----TFCGKN--------GIIFEDGEWWSIENQTESD-------

DURV_G KVTIKYKLS--YRHSKAYGY----------------ISGPAVPFSDL--RDACKI-----KFAGTA--------GMRLGSGMWISLGGDHEQGGLG----

FLAV_G TWETGKVFG--VLEEKLYQNGYLKDNRFGID--EQWIRSSIYGLRSL--VGSCYR-----GVCRQF--------GIRFKTGEWWGLEGKDVTG-------

IHNV_G Q-EIKGHLFVDKISNRVVK-----------------ATSYGHHPWGL--HRACMI-----EFCGGK--------WIRTDLGDLISVEYNSG---------

NGAV_G HWEHSKIYALTGESSEVLKDDESYDYRYKELIYLSFLESGSYGMRST--KNACKT-----DICGVK--------GIRFSTGEWWGIVDREKT--------

RABV_G T-NSRGKRA--SKGSETCG----------------FVDERGLYKSL---KGACKL-----KLCGVL--------GLRLMDGTWVSMQTSNET--------

TUPV_G F-DETMVTV--TVEAKKVVK-------------DSWVTGATITPSVM--EGSCKK-----TLGSKS--------GILLPNGQWFSIVETGQITIQPKGSV

VSINV_G L-ISMDITF--FSEDGELSS-------LGKEGTG--FRSNYFAYETG--DKACKM-----QYCKHW--------GVRLPSGVWFEMADKDL---------

WONV_G AWEYSHVFA--DIDIDHNNNHPI----YSIGKT---IDSEIYGPRDL--ADACII-----KICGIP--------GIRFSHGEWWGIKTLSDRI-------

TIBV_G TWECHPIKI--YYGWVSKKKNQETSTTFNYVQTGLVIESQYIGHVLM--ADLCIM-----TFCNRD--------GYLFPDGSWWEIKYSLYHAFTKDH--

MOUV_G --ENEKAVL--YVEKQNVVP-------------VVWVKLTGGTVYKL--DRACTM-----TYCDID--------GVRMEDGHWFAGVNLTQYVR------

DMeSV_G Q-QSMGLIY--SSRLYGERE-------PMLEVGS--IHIEGHRDKNL--TLACRI-----SFCGEI--------GVRFHDGEWMKVSVNLDHPNSVT---

NIAV_G SWTNIYAKY--KRVGPHMGDWK--------------FWGGGMPTSTF--KDACKM-----EFRGKE--------GIRVSSGFWFHIPQMDDVEFK-----

FARV_G ELVLVDLNPLSPG-HGGYAVLLPNGDKVDVHDKHAWDGDNKMWRWVYEKKDPCAFELVSREVCLFSLSRGSRLRGATPPQGELLTCPHSGKAFDLKGARR

**a b IX X ***

BEFV_G --------FQNFKI-EKCK----GKKPGFRMHTDRTEFEELDIKAELEH--------------------------------------ERCLNTISKILNKE

DURV_G WSSKHTTNIFNDLQIDDCAP---GTKVSVPHAGHIGDLLEIKMNHMAMQ--------------------------------------FLCLDALRNVFREN

FLAV_G --------WIKQII-PRCQE---NQYVSFHHDNSD--------ENIAEA--------------------------------QLVARELVCEEFLGRAKGGD

IHNV_G --------AEILSF-PKCE----DKTVGMRGNLDDFA----YLDDLVKA----------------------------------SESREECLEAHAEIISTN

NGAV_G --------YLEVAF-PDCTP---NIDITLHHLHSG-------SSHIYEH---------------------------------NVFKDYHCKDVISRLMSGA

RABV_G ---------------KWCPP---DKLVNLHDFRSD------EIEHLVVE--------------------------------ELVRKREECLDALESIMTTK

TUPV_G EEKETWVNLINDLNLSDCAE---TQEAKVPTAEFT------VYKTESMV--------------------------------FNILNYHLCLETVAKARSGK

VSINV_G --------FAAARF-PECPE---GSSISAPSQTSV------DVSLIQDV--------------------------------ERILDYSLCQETWSKIRAGL

WONV_G --------PLEDII-IKCHN---GTSVGFVHNIW-------TPSELVGE---------------------------------ITYRDHKCLDVLSSFLGQR

TIBV_G --------TVLNNA-HKCGDRTH-DHLTEFQRDKKVGYEDLEINLEGLE-----------------------------MRQKSRSINMMCLNRLAEIRNTH

MOUV_G ---------------RNCDK---GMDITFDTLASL------SLLTKIEL--------------------------------EHVQDRMECLDAVQDLRAGG

DMeSV_G --------FQVTDF-PPCPP---GTTIQTAVVENINP----EIQELTVN----------------------------------MMYRLKCQETISKMVSGL

NIAV_G --------TEYGKL-AHCVS---SKEIKFPSAHEEVAEHEMEIQDLILT--------------------------------------LRCRDIIDKYEETG

FARV_G ------------ITPISCKIDMEYDLLSLPTGVILGLHLSELGTSFGNLSMSLEMYEPATTLTPEQINFSLKELGSWTEAQLKSLSHSICLSTFSIWELS-

**XI XII**

BEFV_G NINTLDMSYLAPTRPGRDYAYLFEQTSWQEKLCLSLPDSGRVSKDCNIDWRTSTRGGMVKKNHYGIGS-----YKRAWCEYRPFVDKNED----------

DURV_G STSRLDLAKLSPSDAGEHPVYQ-----------------------------------LS------ERG-----IEVGITMYGLINWAPDA----------

FLAV_G LISPFDLNYLLPLNPGLGPSYR--------------------------------AFKRILKKDSHGGSSPQFRLEKRDCIYSVVHNVTEK----------

IHNV_G RVTPYLLSKFRSPHPGINDVYA-----------------------------------MH------KGS-----IYHGMCMTVAVDEVSKD----------

NGAV_G KVSPTDISLLVPDQPGIGHAYK------------------------------IDIVGARNTQNQNSPVPYSLQFKQRVCLYQLIDTTGQS----------

RABV_G SVSFRRLSHLRKLVPGFGKAYT-----------------------------------IF------NKT-----LMEADAHYKSV----------------

TUPV_G NLTRLDLARLAPEIPGVAHVYQ-----------------------------------LT------SDG-----VRVGSTRYEIIAWKPTM----------

VSINV_G PISPVDLSYLAPKNPGTGPAFT-----------------------------------II------NGT-----LKYFETRYIRVEIAAPI----------

WONV_G KINPYELSYLVQDFPGEGPAYR-----------------------------------IMKQYTGNNKTKATFRLQTKTCRYHVAYIDKLT----------

TIBV_G HINVLDMSYLTPKHPGRGLAYY-----------------------------------FS----QDQKNSSKYHVKVLDCDYKLIHIHDADIKGFVNITKY

MOUV_G KVTYAKLSKLQPRRGGLFHVYR-----------------------------------IN------KGT-----LEYTMGRYEGLTSLITN----------

DMeSV_G PTSALDLSYLIQVQEGPGIVYK-----------------------------------RE------KGV-----LYQSVGMYQYIDTVT------------

NIAV_G SISFMDLALFDPDNEGPAHIYR-----------------------------------IN------KGK-----LEAGLVNYGECKVSKKG----------

FARV_G ------VGMID-LNPTRAARALLHD--------------------------------DNILATFENGH-----FSIVRCRPEIVQVPSHP----------

**a b e**

BEFV_G --------------------GYIDIQELNGHNMS----------------------GNHAILE------------------TAPAG-----GSSGNRLNV

DURV_G ----------------RENRLGYHYGTSKAEGKN------------------------PITWK---R--------------WTRTQ-------DGKLNG-

FLAV_G --------------------VNITNNKLAI-GQLFDGSYVYINESEFSRPDYLNNSDNASRDD------------------WFLLS--------------

IHNV_G ---------------------RTTYRAHRA--------------------------ISFTKWE-------------------RPFG--------DEWEG-

NGAV_G ----------------FNVEGNPEGGRVKV-GVSHSGGDVYINISQFEATMTGQREVADSGDEQVLI--------------WTAEG--------TKTYS-

RABV_G ----------------------RTWNEILP-SKG----------------------CLRVGGR--C---------------HPHVN--------GVF---

TUPV_G -------------------GLDKTLGLTIVPSGN--------------------RNSETIKWI---E--------------WTRTD-------DGLLNG-

VSINV_G --------------------LSRMVGMIS--GTT----------------------TERELWD---D--------------WAPYE--------DVEIG-

WONV_G --------------------FDPENGTDEVYKLG------TWGNGRTVILNSTEVGINPTYINKSFD--------------WEPLE--------------

TIBV_G PEPNVTILGLKDNLTFADLGISRCQDLTPLNGSR---NISCEESSGPLHSDDSRLSNGKRFWT------------------RHSFQGANFHEHPGVRIG-

MOUV_G --------------------IPFVIGKNQK--------------------------DEKVRL----P--------------HIPSG------DNSTLSS-

DMeSV_G --------------------LNTEENQLGENARG-----------------------QKVFWT---E--------------WSDSP-----TRPDLQEG-

NIAV_G --------------------DPAESACVKVMDNG---------------------QRSPIFFQ---D--------------WVPTG------IKGIQSG-

FARV_G ---------------------RACHMDLRPYDKQSRASTLVVPLDNSTALLVPDNIVVEGVEASLCNHSVAITLSKNRTHSYSLYP--------QGRPVL

**c d**

BEFV_G TLNGMIFVE----PTKLYLHTKSLYEGIEDYQKLIKFEVMEYDNVEENLIRYEEDEKF--KPVNLNPHEKSQINRTDIVREIQKG-GKKVLSAVVGWFTS

DURV_G -PNGVYQS-----NNTIVHPNLALLGNLIMEDLAADFDLDPIKEPEVTHYDANWETES-------------------IGRGEDTR-YMTHRNRFFFW---

FLAV_G -LNGMIKY-----GNSVYLPHGVSTGLSGIQDIVERGTLMLLDHPKSIAISNQMDLAK------NIYTSYFQMNTTSIGSKIENM-IIRAKNAVSSYFS-

IHNV_G -FHGLHG------NNTTIIPDLEKYVAQYKTSMMEPMSIKSVPHPSILAPYNETDVSG---------------ISIRKLDSFDLQ--------SL-----

NGAV_G -INGMYLRTKNNTPTQLIFPSSAMLEGLYDESLLYPVSLDILQRPRVLIVGPQDDYVK------KILENDNTLNRTDIIEGTKKA-INSFVDKITGVF--

RABV_G -FNGIILGP----DGNVLIP-EMQSSLLQQHMELLESSVIPLVHPLADPSTVFKDGDE-----------AEDFVEVHLPDVHNQV-SGVDLG-LPNWG--

TUPV_G -PNGIFIAD----GKEIVHPNLKMVSFELETYLISEHSTQLVPHPVIHSISDEIYPEN------------------YTIGGKNSY-IKIHTPTAYFWSGI

VSINV_G -PNGVLRT-----SSGYKFPLYMIGHGMLDSDLRLSSKAQVFEHPHIQDAASQLPDDE--------------TLFFGDTGLSKNP-IELVEGWFSGW---

WONV_G TFNGLMRF-----GAELVLPQAVYTDHPNITNLLEDYEISLIGHPKEIFEPEQDELSQ-------VYKFYERSNSTNVVGLASNF-VKTIGRSIGNFFGG

TIBV_G -VNGITYDIR---KQILRFPSTSNLLWDLPSYYSTKHRVHFFQHPTKHEIRKNFTGSDSRDIDVLDDLINRHINRTDFPTRIRNW-IGNIEDKVEHFFSN

MOUV_G -YNGVHMFL----NGTVIIPEMELYKLRYSETLLYEHLLGEMKHPSAKQRERMGLTPD------------------DDKRTTNKS-LNIGEWFTSFW---

DMeSV_G -INGIVKY-----EGQIRVPLGMSLRLEAATELMWGHPVHTVSHPILHVISNHTEQSV---------------TTWNRGVNSTNL-IGLATRSISGFYND

NIAV_G -FNGLYRE-----NGEIKHAGYNLFQNKLTESDIQRMELTPIHHPVLLSLSDVAPGLN---------------VTFDQTGERGELDLDLLPGITGIWR--

FARV_G RQKGAVELP-------TIGPLQLHPATRVDLYTLKEFQEDRIAHSRVTDIKAAVDDLR---------------AKWRKGKFEADTTGGGLWSAIVGVFSS

**.***

BEFV_G TAKAV---R-WTIWAVGAIVTTYAIY--------------KLYKMVKSNSSHSKHREADLEGLQSTTKENMRVEKNDKNYQDLELGLYEEIRSIKGGSKQ

DURV_G GPWNSL--K-NILLASIITLIALITSTLLLCCVC------K-------------------------KRQHRSV---------------------------

FLAV_G --------Q-LTNIAWWIGTGILGLLGFIVI---------KRFHLIQLICG------------KKHRNGKIKKKNNKLNNDDQEAHVYDTIFNTPKTPPH

IHNV_G --------H-WSFWPTISALGGIPFALLLAAAACYCWSG-R--------------------------------PPTPSASQSIPMYHLANRS--------

NGAV_G --------K-GFTQIIWWGITGVCTIILW-----------KLYR------------------KYKAWKRNKTTKRTTPNNAKKSTTIYKNPTFREDVESQ

RABV_G --------K-YVLLSAGALTALMLIIFLMTCC--------R---------------------RVNRSEPTQHNLRGTGREVSVTPQSGKIISSWESHKSG

TUPV_G HWIEGAVQK-LFIVVVATALIGLFILVVWLCCGCCS----K--------------------------SRPVRNQKWE-----------------------

VSINV_G --------K-SSIASFFFIIGLIIGLFLVL----------RVSIYLCIK-----------------LKHTKKRQIYT----DIEMNRLGK----------

WONV_G T-------K-NLIWWLVTVALSTIGTYIAY----------KLGLFKFLG-------------RILFQGSESKEDKRVSNIYEEPLKLGGRRSHLVKNPFF

TIBV_G VGGTI---K-TIISLVLFVIGTLISI--------------KVWKKCKR------------HPQKTKKVAQLKLNDYEKTYNQRDTSNNNNDDLYETIENG

MOUV_G -SHLVG--K-IVSILGTALAIFLILYICWICL--------K----------------------IQIKRVSDKNRVDQMEMQILSKARAPEVRPTLSGPIW

DMeSV_G L-------K-LYLILALIVVSIVALVVLDVIPF-------KYILFILCPP-----------LLLCRFIKCSRRRPETGDRYHVEYNRPGQVSSAF-----

NIAV_G --------K-FVEYLSMAALILTLIVSIFVVW--------KCCI----------------------------SNHLGPSKKTSEMEYFE-----------

FARV_G LGGFFM--R-PLIALAAIVTSIIILYILL-----------RVLCA-----------------ASCSTHRRVRQDSW------------------------

BEFV_G TGDDRFFDH----------

DURV_G -------------------

FLAV_G GKGTGVKYFDY--------

IHNV_G -------------------

NGAV_G SEHLYDSVDMRRPQTYFST

RABV_G GETRL--------------

TUPV_G -------------------

VSINV_G -------------------

WONV_G DNGI---------------

TIBV_G GTVYSPFHV----------

MOUV_G -------------------

DMeSV_G -------------------

NIAV_G -------------------

FARV_G -------------------

1. Walker PJ, Kongsuwan K: **Deduced structural model for animal rhabdovirus glycoproteins.** *J Gen Virol* 1999, **80 ( Pt 5):**1211-1220.
